# Estimating Respiratory and Heart Rates from the Correntropy Spectral Density of the Photoplethysmogram

**DOI:** 10.1371/journal.pone.0086427

**Published:** 2014-01-22

**Authors:** Ainara Garde, Walter Karlen, J. Mark Ansermino, Guy A. Dumont

**Affiliations:** 1 Electrical and Computer Engineering in Medicine Group, The University of British Columbia and BC Childrens Hospital, Vancouver, British Columbia, Canada; 2 Anesthesiology, Pharmacology and Therapeutics, The University of British Columbia and BC Childrens Hospital, Vancouver, British Columbia, Canada; University of Adelaide, Australia

## Abstract

The photoplethysmogram (PPG) obtained from pulse oximetry measures local variations of blood volume in tissues, reflecting the peripheral pulse modulated by heart activity, respiration and other physiological effects. We propose an algorithm based on the correntropy spectral density (CSD) as a novel way to estimate respiratory rate (RR) and heart rate (HR) from the PPG. Time-varying CSD, a technique particularly well-suited for modulated signal patterns, is applied to the PPG. The respiratory and cardiac frequency peaks detected at extended respiratory (8 to 60 breaths/min) and cardiac (30 to 180 beats/min) frequency bands provide RR and HR estimations. The CSD-based algorithm was tested against the Capnobase benchmark dataset, a dataset from 42 subjects containing PPG and capnometric signals and expert labeled reference RR and HR. The RR and HR estimation accuracy was assessed using the unnormalized root mean square (RMS) error. We investigated two window sizes (60 and 120 s) on the Capnobase calibration dataset to explore the time resolution of the CSD-based algorithm. A longer window decreases the RR error, for 120-s windows, the median RMS error (quartiles) obtained for RR was 0.95 (0.27, 6.20) breaths/min and for HR was 0.76 (0.34, 1.45) beats/min. Our experiments show that in addition to a high degree of accuracy and robustness, the CSD facilitates simultaneous and efficient estimation of RR and HR. Providing RR every minute, expands the functionality of pulse oximeters and provides additional diagnostic power to this non-invasive monitoring tool.

## Introduction

The ability to track multiple vital signs from a simple, low cost, and easy to use non-invasive sensor is desirable to facilitate physiological tele-monitoring. There is a clear need for reliable and simple methods for tracking cardio-respiratory activity over time to monitor patients in the intensive care environment or patients at home with long-term disease with associated instability in respiratory or cardiovascular function. Therefore, the remote and automated monitoring of heart rate (HR) and respiratory rate (RR) is an important field of research [1].

An abnormal RR is often an early sign of critical illness. For example, an essential criterion integrated in guidelines for the diagnosis of pneumonia in children (age 1–5 years) is the assessment of an elevated RR (>40 breaths/min) [2]. However, clinical measurement of RR has been shown to have poor reliability and repeatability [3]. A reliable estimate of RR assessed in an automated way is therefore crucial in the application of remote tele-monitoring, where persons with no specialized training are conducting the assessment. This would enable early support for timely recognition and management of physiological deterioration of high-risk patient groups [4].

Pulse oximetry is widely used in health facilities to monitor physiological vital signs. It is based on the principle of photoplethysmography (PPG), an optical technique to measure local variations of blood volume in tissues. Two light-emitting diodes (LEDs) illuminate the tissue and a photo detector detects the light reflected by the tissue. The intensity of the light detected varies with each heart beat as the blood volume changes over time [5]. Blood oxygen saturation (SpO2) is calculated by measuring the difference in absorption of oxygenated and deoxygenated hemoglobin at two distinct wavelengths, red (660 nm) and infrared (940 nm). Oxygenated blood preferably absorbs infrared light and transmits red light and deoxygenated blood has the inverted absorption characteristics [4].

The PPG is a complex signal composed of different but related components. The most recognized PPG waveform component is the peripheral pulse synchronized to each heart beat (AC component). This AC component is superimposed and modulated by a quasi DC component that varies slowly due to respiration, vasomotor activity and vasoconstrictor waves [4]. In addition, an autonomic response to respiration causes a variation of HR synchronized with RR, referred to as respiratory sinus arrhythmia. The PPG signal is also influenced by other mechanisms that are not completely understood. However, it is generally accepted that it has potential to provide clinically useful information about the cardio-vascular and respiratory system [6] and its SpO2 pattern characterization has successfully applied to detect sleep apnea [7].

Well-established methods have been described for the estimation of SpO2 and HR from the PPG [8], [9]. In addition, several methods based on characterization of the PPG cycles morphology in the time domain, using time-frequency analysis [10], [11], [12], [13], [14], [15], [16] digital filtering [5], [17] and smart fusion [6] have been proposed to estimate RR. However, this estimation of RR in pulse oximetry is not yet commercially established. The simultaneous estimation of HR and RR from the PPG signal would provide a low processing overhead that is desirable for simple and low cost physiological monitor. This would reduce vital sign monitoring hardware to one peripheral sensor and one signal-processing step.

Correntropy-based spectral density (CSD) has been found to be particularly well suited for the characterization of modulated signals. This method provides an improved spectral resolution compared to conventional techniques like power spectral density (PSD)and shows promise in the detection of modulated patterns [18]. Correntropy is a generalized correlation function that provides information on higher-order statistics. It is able to detect nonlinearities that conventional techniques (based on second-order statistics), may be unable to detect. Another attractive property of the correntropy function is its robustness against impulsive noise [19], [20].

In this paper we propose a novel algorithm based on CSD to estimate both RR and HR simultaneously from the PPG signal obtained from pulse oximetry. The initial application will be to develop an easy-to-use portable device that measures multiple vital signs. This algorithm is ideally suited to be implemented on the *Phone Oximeter*®, a mobile device that integrates a commercially available and federal drug administration (FDA) approved pulse oximeter (Xpod) with a mobile phone. The *Phone Oximeter*® enables the analysis of vital signs and intuitive display of information to health care providers [21]. In addition, *Phone Oximeter*’s SpO2 characterization has been successfully applied to detect sleep apnea [7].

This paper is organized as follows; the Materials and Methods section describes the dataset used for the development and testing of the newly developed algorithm to estimate RR and HR based on CSD, and explains the algorithm with brief description of CSD and PSD methods. The accuracy of the CSD-based algorithm is presented in the Results section, which is followed by the Discussion, Limitations and Conclusion sections.

## Materials and Methods

### CSD-based Algorithm

**Figure 1:**
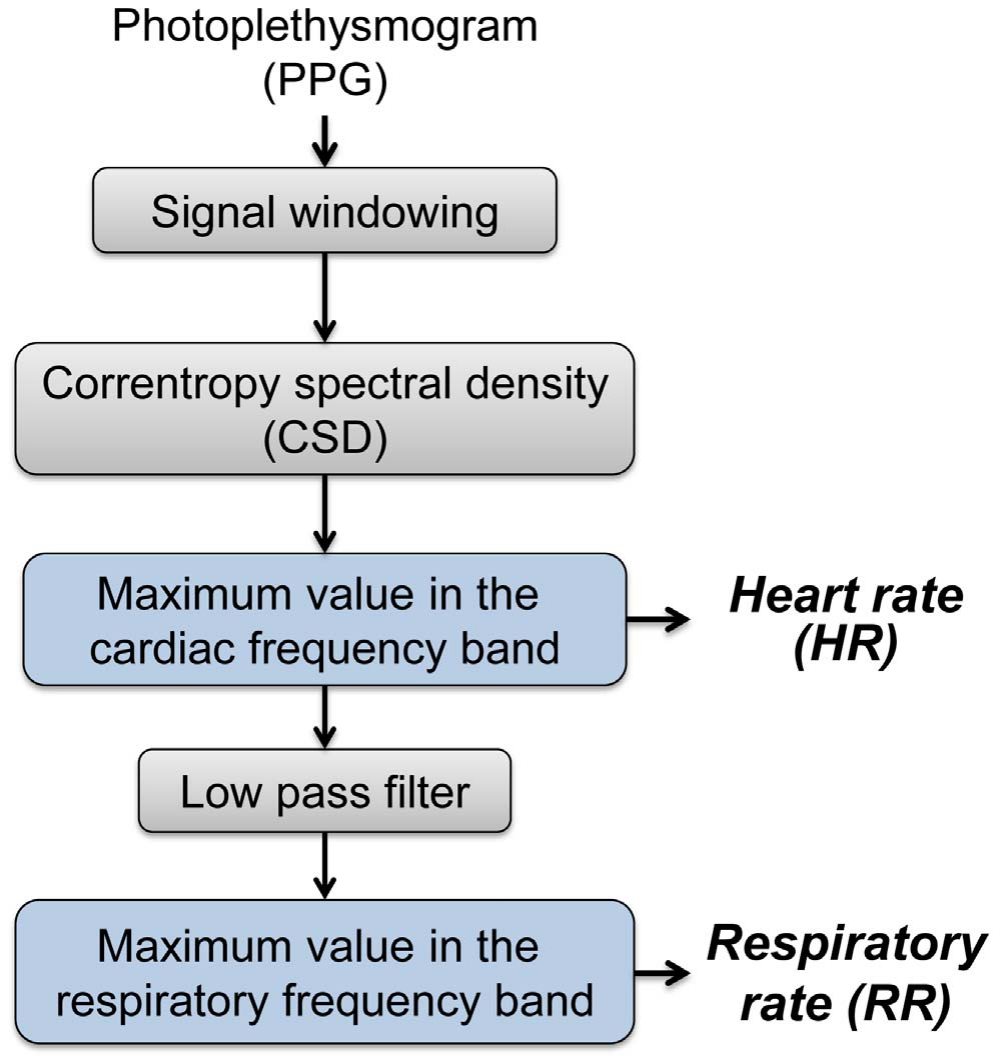
Overview of the CSD-based algorithm. Initially the PPG signal is segmented into windows (60 s or 120 s) with 50% of overlap. In the subsequent step the CSD is applied to calculate the spectrum of the windowed signals. The HR is estimated by detecting the maximum frequency peak within the cardiac frequency band. The signal is then low pass filtered and the RR is estimated by detecting the maximum frequency peak within the respiratory frequency band.

Conventional spectral analysis assumes a stationary signal and is therefore unable to identify HR and RR changes over time. An approach to account for such changes is to implement a time-varying spectral analysis. Firstly, a sliding time window of 60 s or 120 s with 50% overlap is used to segment PPG signal into segments assumed to be stationary and suitable for spectral analysis. Secondly, the CSD is applied to the signal segments. Thirdly, the HR is estimated by detecting the maximum frequency peak within the cardiac frequency band and filtered from the signal, and lastly the RR is estimated by detecting the maximum frequency peak within the respiratory frequency band (see Figure 1).

#### Correntropy spectral density

The CSD is a generalization of the conventional power spectral density. It is based on the Fourier transform of the centered correntropy function [18],
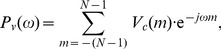
(1)where 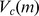 is the centered correntropy function, in which the mean of the transformed data is subtracted so as to reduce the effect of output DC bias. It is estimated by 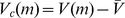 where 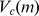 is the correntropy function and 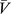 the correntropy mean, defined as:



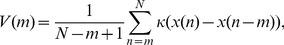
(2)

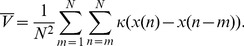
(3)


The sigmoidal, Gaussian, polynomial, and spline kernels are the most commonly used symmetric positive definite kernels, applied to machine learning, function approximation, density estimation, and support vector machine classification [22], [23]. The Gaussian kernel function, applied in the present study, is given by
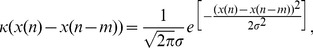
(4)where 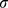 is the kernel parameter, here set using Silverman’s rule of density estimation [19].

Correntropy, introduced by Santamaria et al. [19], is a similarity measure defined in terms of inner products of vectors in a kernel parameter space. It provides information on both the time structure and the statistical distribution. In addition, the use of kernel methods makes the correntropy computationally efficient since it can be computed directly from the data.

Autoregressive (AR) spectral analysis based on the Yule-Walker method was applied to improve spectral resolution compared to conventional techniques [18]. The autoregressive coefficients were estimated from the correntropy function, using the YuleWalker equations [24]. The selection of model order is a trade-off between the frequency resolution and the spurious peaks. The optimal model order between 5 and 15 was selected using the minimal description length criteria defined by Rissanen [25].

#### HR and RR estimation

**Figure 2:**
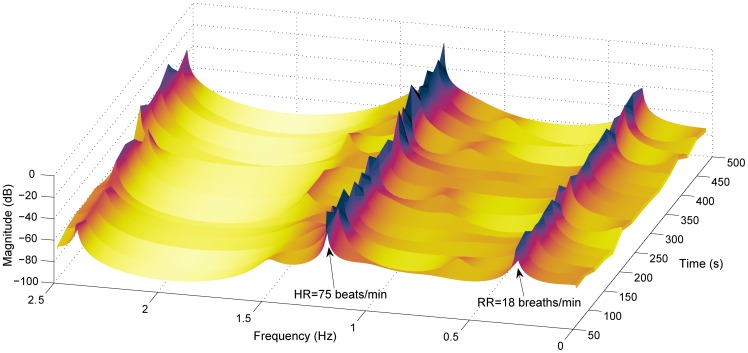
Time-varying CSD of 8-min PPG signal. Both respiratory and cardiac frequency peaks reflect RR and HR, respectively. Respiratory frequency peak is around 0.3 Hz (18 breaths/min) and cardiac frequency peak around 1.25 Hz (75 beats/min).

The CSD over time shows both respiratory and cardiac frequency peaks reflecting the RR and HR respectively (Figure 2). These peaks can be tracked in the region of the respiratory and cardiac frequency bands. Reference HR and RR ranges were extracted from a review of observational studies that used HR data from 143,346 children and RR data from 3,881 children (from 6 months to 18 years old) [26]. Based on 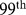 and 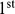 centiles for children and young adults, the HR could range from 30 to 180 beats/min (0.5 to 3 Hz, respectively) and RR from 8 to 60 breaths/min (0.14 to 1 Hz, respectively [26]. The range in adults is much more restricted but would be included in this range. An extreme range may occur in critical illness, such as an elevated HR in the presence of an arrhythmia or an elevated RR (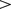 40 breaths/min in children with pneumonia [2]) as an early indicator of critical illness. However, those pathological or abnormal RR and HR values will also be included in this extended HR and RR ranges extracted from the review. Therefore, the maximum value peak frequencies in the cardiac frequency band (0.5 to 3 Hz) and in the respiratory frequency band (0.14 to 1 Hz) were automatically extracted, reflecting HR and RR, respectively.

**Figure 3:**
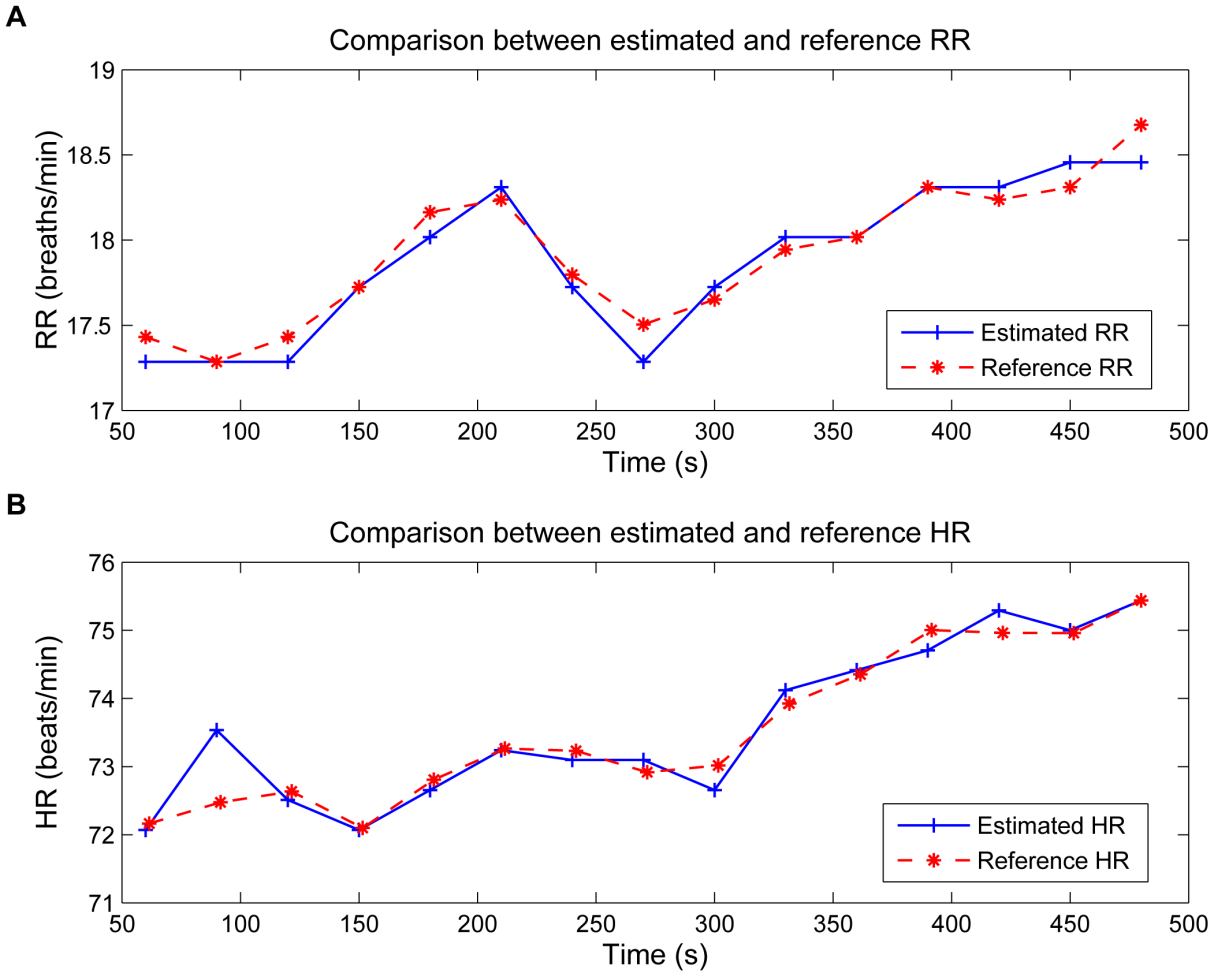
Time-varying estimated and manually labeled reference RR and HR. Estimated (solid blue with * markers) and manually labeled (dotted red with+markers) reference RR in (A) and HR in (B). For this subject the RMS errors estimating RR and HR are 0.25 breaths/min and 0.35 beats/min, respectively.

For improved resolution around the respiratory frequency peak, the HR was filtered using a zero-phase 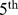 order low pass filter with a cutoff frequency of 0.1 Hz below the cardiac frequency. In addition, frequency peaks close to the secondary harmonics around HR were excluded when an elevated RR (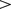 45 breaths/min) were detected. An example of the RR and HR extracted from the time varying CSD (Figure 2) is illustrated in Figure 3.

#### Power spectral density

Following the same concept a PSD-based algorithm was implemented. For a parametric PSD, the signal 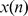 was modeled through an AR model by
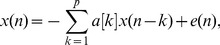
(5)where 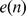 denotes zero-mean white noise with variance 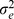, 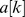 the AR coefficients and 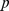 the model order. Once the autoregressive coefficients and the variance 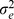 have been estimated, the PSD of an autoregressive process is computed by means of



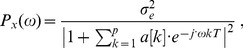
(6)being 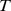 the sampling period. As for the CSD, the optimal model order between 5 and 15 was selected using the minimal description length criteria defined by Rissanen [25].

### Simulation Database

To show certain performance properties of the algorithm a simulated PPG signal was first produced. Respiration has three different effects on the PPG waveform. The first and more predominant effect is a shift in the baseline during each breath. The second is a change of the amplitude of the pulse beats with each breath which implies that the PPG signal is subject to amplitude modulation (AM) [15]. The third effect is a variation of HR due to an autonomic response to respiration and usually decreases with age. Based on the first 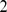 effects for sake of simplicity, the PPG signal was simulated using AM and a baseline shift as follows:
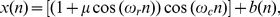
(7)where 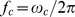 is the cardiac frequency, 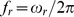 is the respiratory frequency, 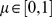 is the modulation index and 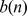 is the baseline shift synchronized with 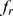. One hundred outliers with values between mean 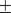 5 standard deviation of 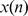 were randomly added to the signal to simulate noise.

### Capnobase Database

#### Ethics statement

All subjects were studied according to a protocol approved by the University of British Columbia and Children’s and Women’s Health Centre of British Columbia Research Ethics Board. Informed and written consent to be part of the research database was obtained for all subjects. For subjects under 16 years of age, parental/guardian written consent was obtained. Written assent was obtained for all subjects over the age of 11 years.

#### Database

Capnobase is an on-line database that contains physiological signals collected during simultaneously elective surgery and routine anesthesia for the purpose of development of improved monitoring algorithms in adults and children [27]. The signals were recorded from 59 children (median age: 8.7, range 0.8–16.5 years) and 35 adults (median age: 52.4, range 26.2–75.6 years) receiving general anesthesia at the British Columbia Children’s Hospital and St. Paul’s Hospital, Vancouver BC, respectively. The recordings included ECG with a sampling frequency of 300 Hz, capnometry with a sampling frequency of 25 Hz, and PPG with a sample frequency of 100 Hz. All signals were recorded with S/5 Collect software (Datex-Ohmeda, Finland) using a sampling frequency of 300 Hz (PPG and capnometry with lower sampling rates were automatically up-sampled).

Capnobase contains a benchmark dataset with forty-two 8-min segments from 29 pediatric and 13 adults cases containing reliable recordings of spontaneous or controlled breathing. The capnometric waveform was used as the reference gold standard recording for RR. A research assistant manually labeled each breath in the capnogram and pulse peak in the PPG and validated the derived instantaneous reference RR and HR. The beginning and end of all artifacts in the PPG waveforms were also manually labeled and almost 50% of the cases contained artifacts due to movements or similar noise. Capnobase also contains a calibration dataset with one hundred twenty-four 2-min segments randomly selected from the remaining 52 cases. This dataset is particularly challenging because it includes other disturbances such as cardiac oscillations etc., which influence the respiratory induced parameters and it also contains substantially more movement artifacts than the benchmark dataset. Signals with significant apnea have been excluded from the analysis. Datasets can be downloaded from the on-line database, CapnoBase.org [27]. CSD-based algorithm was optimized using the calibration dataset and then validated using the benchmark dataset. Both, the calibration and benchmark datasets with reference RR and HR have been previously used to test RR estimation from PPG [6].

### Algorithm Evaluation

The accuracy of the CSD-based algorithm was evaluated and compared to other methods, using the un-normalized root mean square (RMS) error. The RMS error was calculated for each subject, considering all estimations over time:
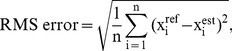
(8)where n is the number of observations and 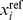 and 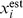 are the reference and the estimated values, respectively. The median of the instantaneous reference RR and HR were compared to the estimations for each time window.

#### Calibration

The spectral resolution increases with longer time-windows with a concomitant reduction in real-time performance (clinicians are required to wait longer for each estimation). To investigate the trade-off in window size, the accuracy of the algorithm was evaluated with the calibration dataset, using time windows of 60 s and 120 s with an overlap of 50%. The statistical significance of the error with the different windows was evaluated using Wilcoxon signed-rank test to compare related samples.

The choice of the kernel parameter (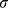) is trade-off between the power of the respiratory peak and the spurious peaks. The power of the respiratory peak and spurious harmonics decreases as 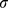 increases [18]. The CSD-based algorithm’s sensitivity to 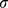 was evaluated using the calibration dataset. The 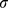 calculated according to Silverman’s rule (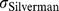) was used as a reference.

#### Validation

The calibrated algorithm was then validated using the Capnobase benchmark dataset. All subjects and all signal segments with mechanical or spontaneous breathing, including those with artifacts, were analyzed. The median error and 1^st^ and 3^rd^ quartiles were calculated to account for a non-normal RMS distribution. A Bland-Altman plot was also performed to compare the estimated HR and RR to the reference rates.

In addition, the performance of our algorithm was compared to previously proposed methods based on PPG cycles morphology [6], time-frequency analysis [15], [7] and digital filtering [17], using the Capnobase benchmark dataset. These methods have been implemented according to the description included on these papers.

A Wilcoxon signed-rank test for related samples using Bonferroni correction for multiple comparisons was also applied to evaluate the statistical significance of our algorithm’s improvement. The normality of all distributions was tested using One-Sample Kolmogorov-Smirnov test.

## Results

### CSD Output

**Figure 4:**
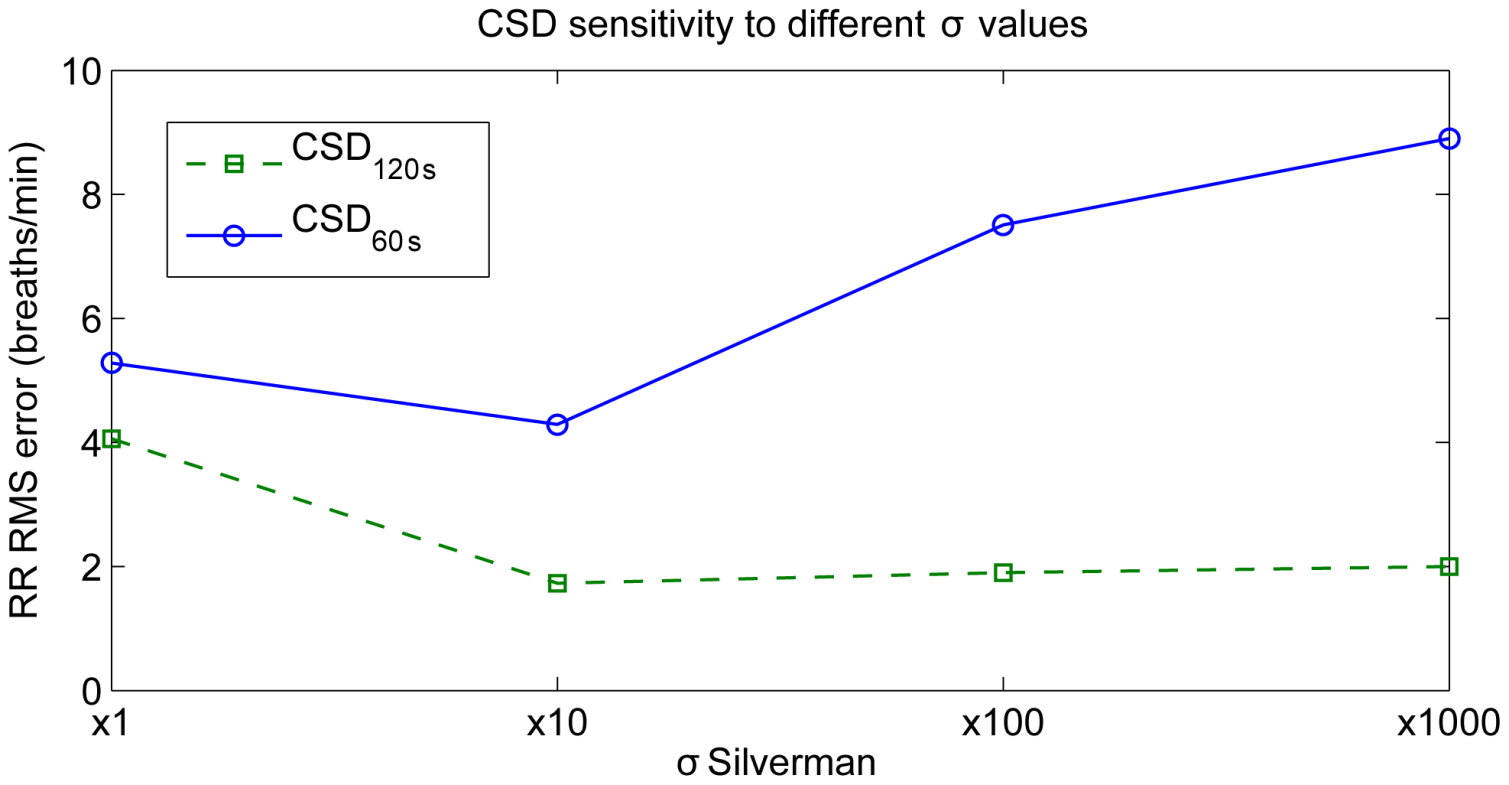
CSD sensitivity to the kernel parameter. CSD-based algorithm’s perfomance estimating RR is illustrated for the kernel values: 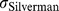, 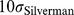, 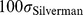 and 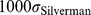. The 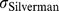 is calculated by Silverman’s rule.

The median RR error obtained with the CSD-based algorithm applied to the calibration dataset was 4.2 breaths/min when using 60-s windows and 1.9 breaths/min when using 120-s windows. The RMS error significantly (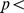 0.05) decreased with longer windows. A kernel size of (10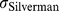) reduced the spurious harmonics and provided more accurate RR estimates (see Figure 4) [19]. Therefore, a 10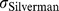 was applied to the Capnobase Benchmark dataset.

**Figure 5:**
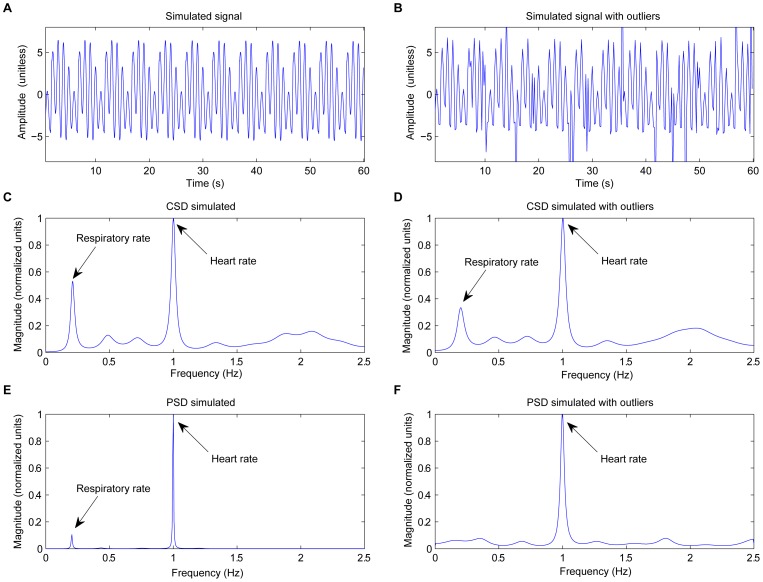
CSD applied to a simulated signal. (A) Simulated signal with 0.2 Hz modulation respiratory frequency (12 breaths/min), 1 Hz cardiac frequency (60 beats/min), and 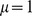, (B) same simulated signal with some outliers randomly added, (C) and (D) the CSD of the simulated signal with and without outliers, and (E) and (F) the PSD of the simulated signal with and without outliers, respectively. CSD analysis provides a clearer and more robust against outliers respiratory frequency peak than conventional PSD.

**Figure 6:**
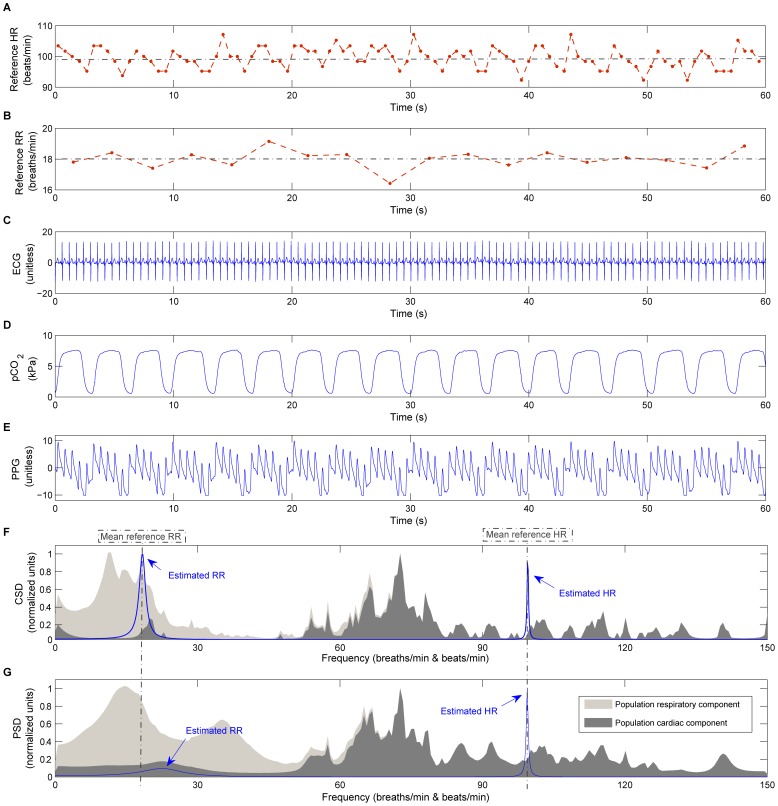
CSD applied to an in-vivo signal. CSD and PSD performance applied to an in-vivo signal (1 min) of one infant subject: (A) reference HR (dotted red line with 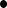 markers) and mean HR represented by a dotted grey line, (B) reference RR (dotted red line with 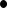 markers) and mean RR represented by a dotted grey line, (C) ECG signal, (D) capnometry, (E) PPG signal, (F) CSD and (G) PSD applied to the PPG signal. In addition, the average CSD and PSD spectrum of the database’s population is illustrated in the background on (F) and (G) respectively, where the cardiac component is represented in dark grey and the filtered signal that corresponds to respiration in light grey.

CSD shows two clear frequency peaks at HR and RR locations, for both simulated and in-vivo signals (Figure 5 and 6). As reported in our previous work [18], the AM effect is reflected in CSD through a frequency peak at its true position. In comparison, the AM in PSD is manifested as secondary harmonics surrounding the cardiac frequency peak. Further, CSD is more robust to impulsive noise (see Figure 5.D and Figure 5.F, respectively) compared to the PSD approach. This is because the Gaussian kernel makes 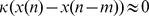 when either 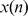 or 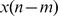 is an artifact.

Although the baseline shift is present in both PSD and CSD, CSD provides a more robust respiratory modulation frequency peak compared to the PSD (see Figure 5.C and Figure 5.E, respectively). The enhanced modulation peak is also observed in the in-vivo signals (Figure 6.F and Figure 6.G, respectively) where the CSD analysis provides a clearer respiratory frequency peak compared to the PSD.

### Benchmark Accuracy Measurements

**Figure 7:**
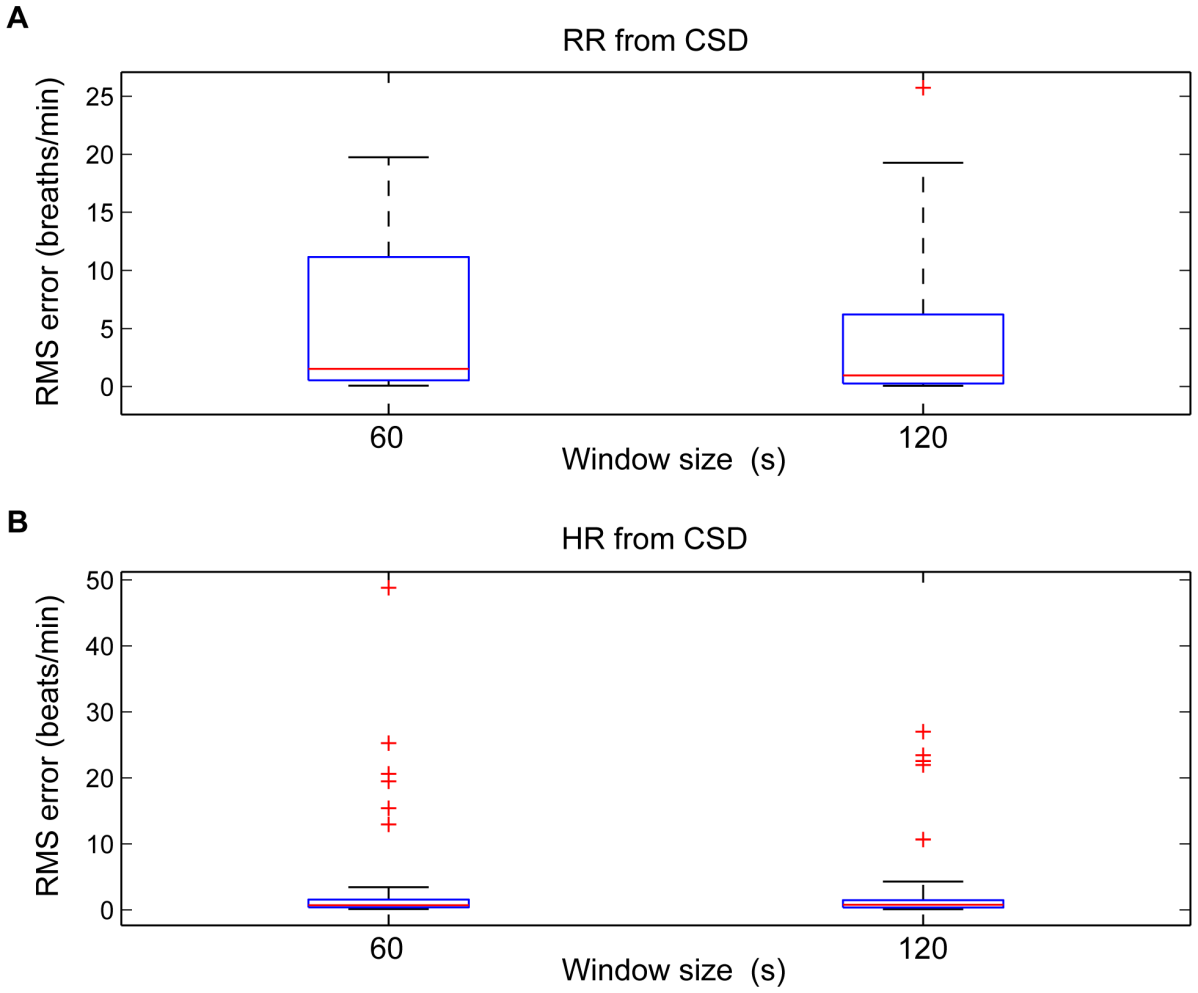
Boxplot of RMS error. Boxplot of the (A) RR and (B) HR RMS error estimated using time windows of 60 s and 120 s and tracked around extended RR (from 8 to 60 breaths/min) and HR (from 30 to 180 beats/min). 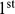 quartile, median, and 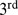 quartile values are displayed as bottom, middle and top horizontal line of the boxes. Whiskers are used to represent the most extreme values within 3 times the interquartile range from the quartile. Outliers (data with values beyond the ends of the whiskers) were displayed as crosses.

The CSD-based algorithm provided a significantly lower RR error compared to the PSD-based algorithm (Table 1). Expanding the permitted cardiac and respiratory frequency bands, increased the total range of the errors (Figure 7). RR or HR misdetections increased the RMS error considerably.

**Table 1:** RMS error estimating RR and HR with different methods.

	Median (1^st^ and 3^rd^ quartile)	
Methods	RR-RMS error (breaths/min)	HR-RMS error (beats/min)
CSD_120 *s*_	0.95 (0.27, 6.20)	0.76 (0.34, 1.45)
PSD_120 *s*_	3.18 (1.20, 11.3)*	0.58 (0.21, 1.17)
Karlen et al. [6]	1.56 (0.60, 3.15)	0.48 (0.37, 0.77)
Garde et al. [7]	3.5 (1.1, 11)*	0.35 (0.2, 0.59)
Shelley et al. [15]	1.91 (0.41, 7.01)	n/a
Nakajima et al. [17]	7.47 (0.59, 10.6)*	n/a

RMS error median (quartiles) estimating RR and HR using different methods. The statistical significant difference (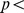
0.05) of the RMS error obtained with the CSD-based algorithm in comparison to other methods is indicated (asterisk *).

The median RR error significantly decreased (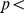 0.05) with longer time windows for using CSD (from 1.77 to 0.95 breaths/min) and PSD (from 7.82 to 3.18 breaths/min) approaches. However, the median error was not statistically different in estimating HR with longer time windows.

**Figure 8:**
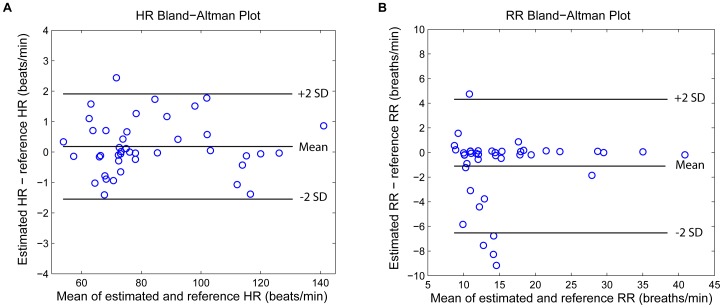
Bland-Altman for HR and RR estimation. Bland-Altman plots for comparison of (A) HR and (B) RR to the reference HR and RR manually labeled by the research assistant. The bias and 95% of limits of agreement are ploted in solid lines. It showed a bias of 0.18 and limits of agreement of −1.52 to 1.91 beats/min for the estimated HR versus reference HR and a bias of −1.1 and limits of agreement of −6.52 to 4.32 breaths/min for the estimated RR versus reference RR.

A Bland-Altman plot (Figure 8) showed good agreement with a HR bias of 0.18 and limits of agreement of −1.52 to 1.91, and a RR bias of −1.1 and limits of agreement of −6.52 to 4.32.

**Figure 9:**
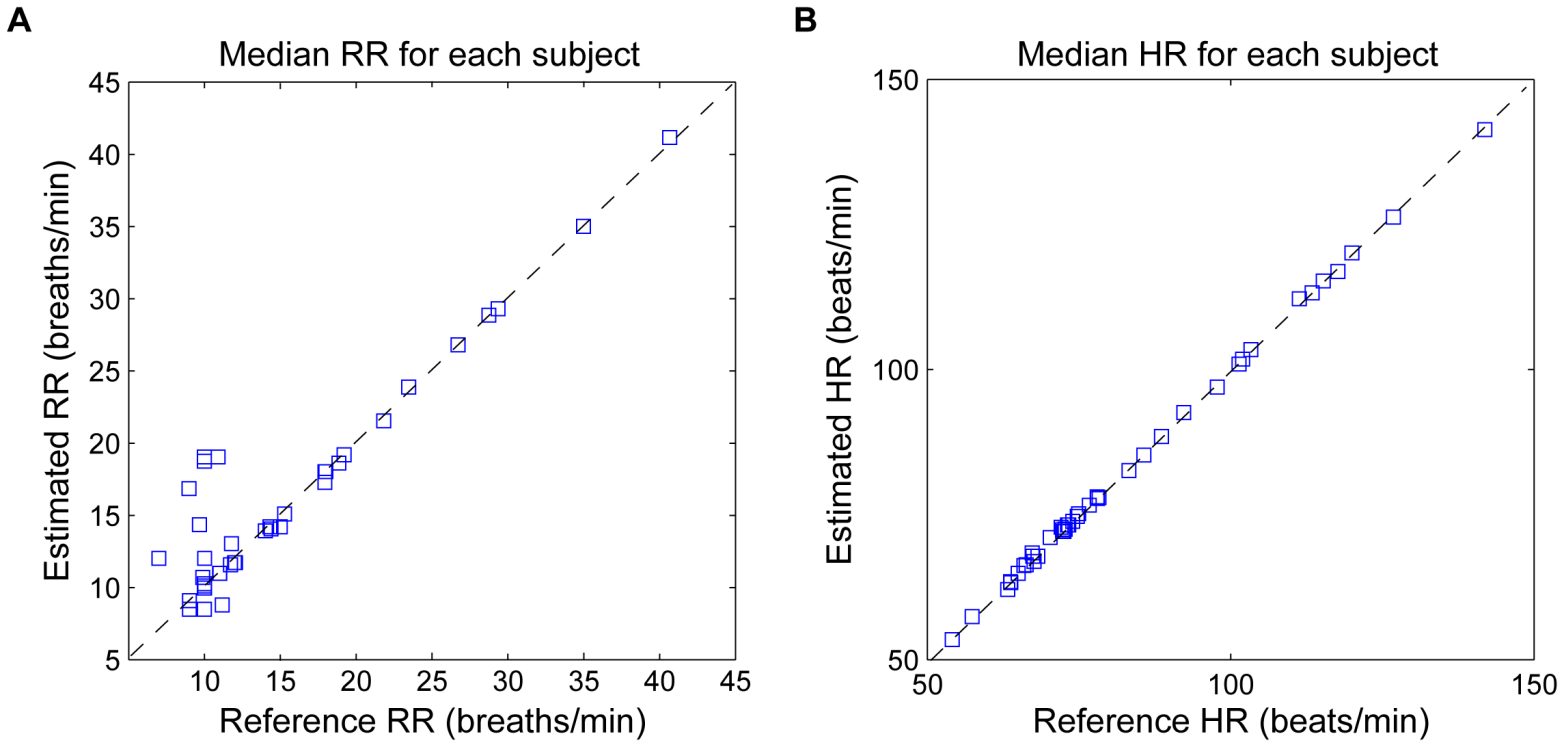
Scatter plot, error per subject. Scatter plot showing the median value of estimated and reference values of (A) RR and (B) HR for each subject using 60-s time window. The respiratory and cardiac frequency peaks are detected around the extended RR and HR range. Observations with artifacts are included. The dotted line represents the optimal performance.

**Figure 10:**
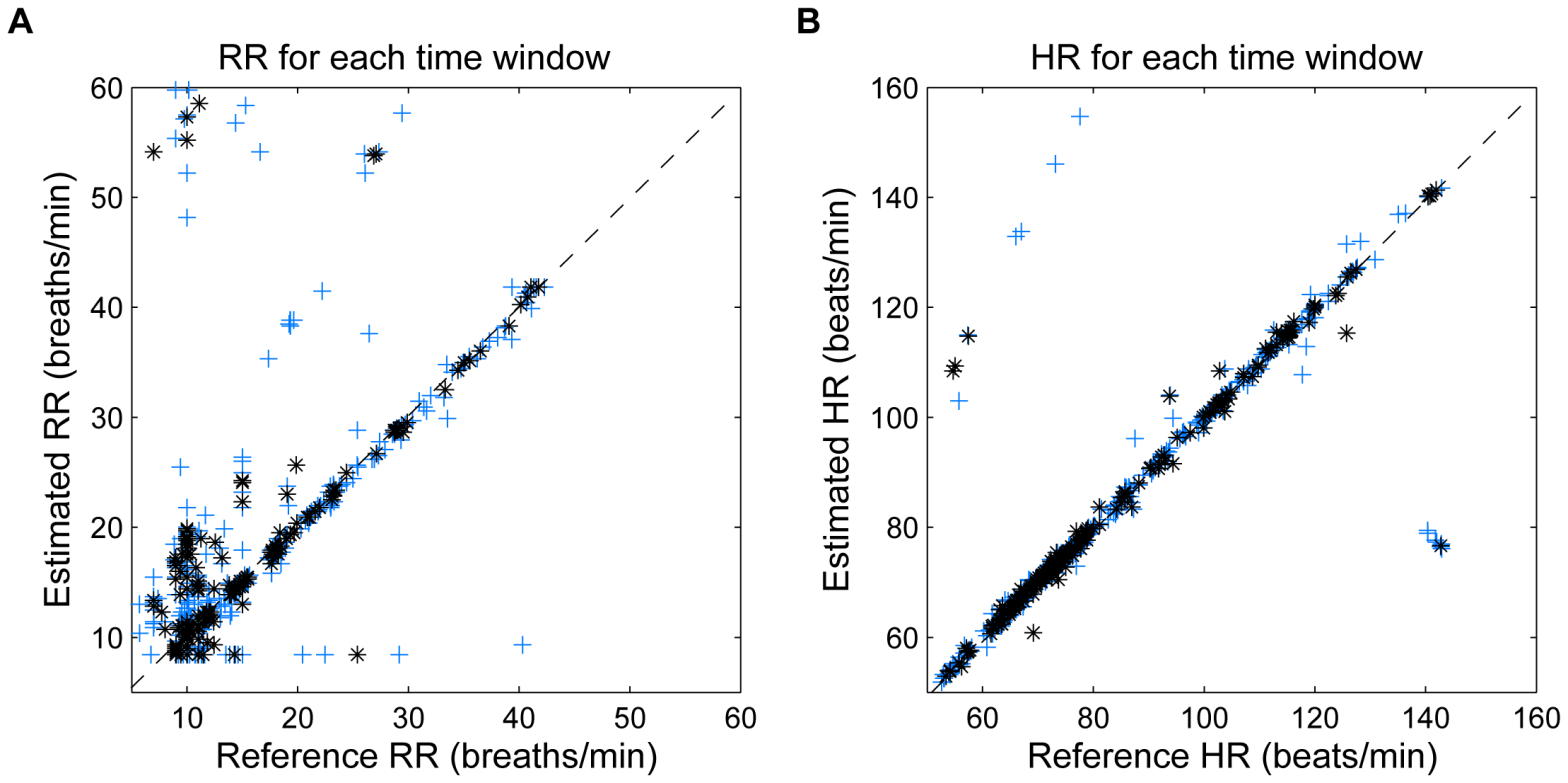
Scatter plot, error per time window. Scatter plot showing the estimated and reference values of (A) RR and (B) HR for each 60-s time window (represented by blue +) and for each 120-s time window (represented by black *). The respiratory and cardiac frequency peaks are detected around the extended RR and HR range. Observations with artifacts are included. The dotted line represents the optimal performance.

The accuracy of the algorithm per subject is illustrated in Figure 9, where the estimated RR and HR using a 60-s sliding window and reference values for each subject are represented. When analyzing the estimation for each time-window (Figure 10), it can be observed that most of RR errors are accumulated at low frequencies (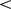 15 breaths/min). However, there are some errors located out of the normal range because of artifacts and the use of extended respiratory and cardiac frequency bands. The number of erroneous estimates was reduced when increasing the window length, which is reflected by a lower error range when using 120-s windows.

Table 1 illustrates the performance of a number of methods using the Capnobase benchmark dataset. CSD-based method provided the lowest RR error when using 120-s windows. A Wilcoxon signed-rank test for related samples with Bonferroni correction for multiple comparisons has shown the significant improvement (p
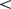
0.05) of our algorithm when compared to PSD and the methods proposed by [7] and [17].

## Discussion

In this study we have presented a novel methodology to estimate both RR and HR simultaneously from pulse oximetry based on CSD. The performance of the algorithm has been validated against a benchmark dataset using RMS error, comparing all estimations with reference RR and HR rates manually labeled by a research assistant. The algorithm has shown high accuracy and robustness estimating RR and HR simultaneously from PPG, even when the search is extended to account for pathological and/or abnormal rates to 8 to 60 breaths/min and 30 to 180 beats/min. In addition to its accuracy and robustness, the RR error significantly decreased when longer time windows were used. Moreover to generalize our findings broad ranges of subjects including children and adults, under controlled ventilation or spontaneously breathing over a wide RR ranges were studied.

Using CSD for frequency estimation is preferable to conventional PSD functions as it accounts for higher-order moments and is more robust to outliers [20]. CSD is particularly useful in signals with amplitude modulation like the PPG signal that was analyzed in this study. CSD provides the modulation frequency at its actual location along the frequency axis [18], instead of at locations of the secondary harmonics surrounding the carrier frequency peak. The respiratory effect on the PPG respiration is usually present as a baseline shift and an AM synchronized with each breath [15]. CSD represents both baseline shift and respiratory AM component at the same position, whereas a direct PSD of the signal provides only the RR derived from the baseline shift at its real position (see Figures 5 and 6). Thus, in signals with a dominant AM component, CSD will provide a more robust respiratory frequency peak.

The results demonstrate that the CSD is an appropriate technique to provide simultaneous and efficient estimation of RR and HR, and will permit to monitor of HR and RR non-invasively using only a peripheral sensor. The relevance of this algorithm from the clinical perspective is that it facilitates an accurate identification of abnormal or pathological rates. Thus, this promising algorithm will expand the functionality and diagnostic power of pulse oximeters. A number of algorithms based on the PPG signal morphology [13], [28] time-frequency or spectral analysis [11], [12], [14], [15], [17] digital filtering [5], [17], and complex demodulation [10] have been proposed to detect RR from PPG. Most of these methods are restricted to healthy ranges only and many are computationally expensive. Moreover, most of these methods have been tested only in controlled environments (research laboratories), and their robustness to artifacts and other influences that are very common in the ambulatory environment have not typically been demonstrated. Therefore, some of these methods have been implemented and applied to the same Capnobase benchmark dataset. CSD-based algorithm has provided lower RR error (0.95 breaths/min) using 120-s windows. The main limitation of the methods proposed by Shelley et al. [15] and Nakajima et al. [17] is that they restrict their estimations to RR 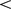 40 breaths/min.

The Smart Fusion method proposed by Karlen et al [6] is computationally efficient and was evaluated in an ambulatory environment. It combines the three respiratory induced variations (frequency, intensity, and amplitude) using a mean calculation. This method improved the robustness of the RR estimation, with a median error of 1.56 breaths/min per subject. However, RR estimation was only provided during periods that have an agreement between the three methods, which significantly reduced the number of estimations. The CSD-based algorithm on the other hand, provides an estimation for each time window. Using the same ambulatory dataset, our algorithm provided a median error of 1.77 breaths/min and 0.69 beats/min per subject, using a 60-s window. Moreover, the CSD-based algorithm showed lower limits of agreement compared to those reported using an acoustic respiratory rate sensor applied to monitor RR in pediatric patients [29].

## Limitations of the Study, Open Questions, and Future Work

Although the algorithm is computationally efficient (around 50 ms per window), the limitation of our algorithm is that a minimum window length of 60 s is required to obtain reliable estimations of HR and RR, whereas the smart fusion could provide RR estimations with segments of 16 s. This requirement reduces real-time performance of the CSD-based algorithm, and decreases the ability to rapidly detect changes such as the onset of apnea that may be critically important in clinical applications. Moreover, despite the promising results, large RR inaccuracies might still occur when dealing with signals with artifacts. Therefore, further research is needed to improve RR estimations. One possible solution may be to combine both morphology-based [6], [30] and CSD-based spectral analysis applied to PPG signal, to provide accurate HR and RR estimations in real time. In addition, we could combine our algorithm with the SpO2 pattern characterization [7] and provide a more robust apnea detector.

Despite its high accuracy on a per-subject basis (Figure 9), the CSD analysis provided some incorrect values when the estimates for each window were analyzed (Figure 10). The errors increased the error range in RR and HR when extended RR and HR are allowed, due to their overlap in the spectral domain (Figure 7). Low frequency processes other than respiration and artifacts can modify the PPG signal and become a confounding factor for accurately assessing RR. Baseline changes, for example introduced spurious peaks at very low frequencies, which distort and shift the respiratory frequency peak, providing more estimation errors at low RR. Due to data windows that contained artifacts, a few incorrect estimations were located out of normal ranges.

For this study the RR and HR were estimated using extended respiratory and cardiac frequency bands when picking the maximum frequency peak (Figure 1). However, these bands could be adjusted or optimized depending on the application. Subjects of different ages, subjects during rest or exercise and critically ill or healthy subjects would dictate the use of different frequency bands. Our CSD-based algorithm detects the respiratory and cardiac frequency peaks from the spectrum. However, including more sophisticated approaches such as an hybrid median filter [31] or optimizing the permitted RR and HR transitions will reduce misdetections due to short time artifacts and baseline changes.

In spite of the promising results of the CSD-based algorithm on the benchmark dataset, its accuracy should be validated with a dataset that includes critically ill children with reduced peripheral perfusion like pneumonia or shock.

## Conclusion

The proposed algorithm estimates both RR and HR directly from the same spectral analysis, without requiring demodulation of the signal or PPG cycle detection and it is more robust to outliers than conventional spectral analysis. Furthermore, it permits the detection of an extended range of possible values that may be associated with pathological clinical states. The implementation of CSD will lead to pulse oximeters that could monitor RR and HR on a per-minute basis with a delay of around 50 ms, expanding the functionality and diagnostic power of this non-invasive monitoring tool. Implementing this algorithm on the *Phone Oximeter*®, will offer the potential for an easy-to-use, intuitive and low-cost tele-monitor that will provide accurate vital signs, especially in low-resource settings. In addition, the combination of both morphology and CSD-based algorithms could provide enhanced HR and RR estimations with higher time resolution.

## References

[1] Olsson E, Ugnell H, Oberg Pa, Sedin G (2000) Photoplethysmography for simultaneous recording of heart and respiratory rates in newborn infants. Acta Paediatrica (Oslo, Norway: 1992) 89: 853–861. 10.1080/080352500750043774 10943970

[2] WHO (2005) Pocket book of hospital care for children. Guidelines for the Management of Common Illnesses with Limited Resources, Geneva, CH. 24006557

[3] Lovett PB, Buchwald JM, Stürmann K, Bijur P (2005) The vexatious vital: neither clinical mea- surements by nurses nor an electronic monitor provides accurate measurements of respiratory rate in triage. Annals of Emergency Medicine 45: 68–76. 15635313 10.1016/j.annemergmed.2004.06.016

[4] Meredith DJ, Clifton D, Charlton P, Brooks J, Pugh CW, et al (2012) Photoplethysmographic derivation of respiratory rate: a review of relevant physiology. Journal of Medical Engineering & Technology 36: 1–7. 22185462 10.3109/03091902.2011.638965

[5] Nilsson L, Johansson A, Kalman S (2000) Monitoring of respiratory rate in postoperative care using a new photoplethysmographic technique. Journal of Clinical Monitoring and Computing 16: 309–315. 12578078 10.1023/a:1011424732717

[6] Karlen W, Raman S, Ansermino JM, Dumont G (2013) Multi-parameter Respiratory Rate Estimation from the Photoplethysmogram. IEEE Transactions on Biomedical Engineering 60: 1946–1953. 23399950 10.1109/TBME.2013.2246160

[7] Garde A, Karlen W, Dehkordi P, Member S, Wensley D, et al.. (2013) Oxygen Saturation in Children with and without Obstructive Sleep Apnea Using the Phone-Oximeter. In: Conference Proceedings - IEEE Engineering in Medicine and Biology Society. 2531–2534. 10.1109/EMBC.2013.6610055 24110242

[8] Allen J (2007) Photoplethysmography and its application in clinical physiological measurement. Physiological Measurement 28: R1–39. 17322588 10.1088/0967-3334/28/3/R01

[9] Lu S, Zhao H, Ju K, Shin K, Lee M, et al (2008) Can photoplethysmography variability serve as an alternative approach to obtain heart rate variability information? Journal of Clinical Monitoring and Computing 22: 23–29. 17987395 10.1007/s10877-007-9103-y

[10] Chon KH, Dash S, Ju K (2009) Estimation of respiratory rate from photoplethysmogram data using time-frequency spectral estimation. IEEE Transactions on Biomedical Engineering 56: 2054–2063. 19369147 10.1109/TBME.2009.2019766

[11] Gil E, Bail R, Laguna P (2011) Deriving Respiration from the Pulse Photoplethysmographic Signal. In: Computing in Cardiology. 713–716.

[12] Lázaro J, Gil E, Bailón R, Mincholé A, Laguna P (2013) Deriving respiration from photoplethysmographic pulse width. Medical & Biological Engineering & Computing 51: 233–242. 22996834 10.1007/s11517-012-0954-0

[13] Leonard P, Grubb NR, Addison PS, Clifton D, Watson JN (2004) An algorithm for the detection of individual breaths from the pulse oximeter waveform. Journal of Clinical Monitoring and Computing 18: 309–312. 15957620 10.1007/s10877-005-2697-z

[14] Orini M, Bail R, Gil E (2011) Estimation of Spontaneous Respiratory Rate from Photoplethysmography by Cross Time-Frequency Analysis. In: Computing in Cardiology. 661–664. 10.1155/2013/631978 PMC3864101 24363777

[15] Shelley KH, Awad AA, Stout RG, Silverman DG (2006) The use of joint time frequency analysis to quantify the effect of ventilation on the pulse oximeter waveform. Journal of Clinical Monitoring and Computing 20: 81–87. 16779621 10.1007/s10877-006-9010-7

[16] Garde A, Karlen W, Dehkordi P, Ansermino JM, Dumont GA (2013) Empirical mode decomposition for respiratory and heart rate estimation from the photoplethysmogram. In: Computing in Cardiology, Sep, 2013. p. In press.

[17] Nakajima K, Tamura T, Miike H (1996) Monitoring of heart and respiratory rates by photoplethysmography using a digital filtering technique. Medical Engineering & Physics 18: 365–372. 8818134 10.1016/1350-4533(95)00066-6

[18] Garde A, Sörnmo L, Jan R, Giraldo B (2010) Correntropy-based spectral characterization of respiratory patterns in patients with chronic heart failure. IEEE Transactions on Biomedical Engineering 57: 1964–1972. 20211799 10.1109/TBME.2010.2044176

[19] Santamaria I, Pokharel PP, Principe JC (2006) Generalized correlation function: definition, properties, and application to blind equalization. IEEE Transactions on Signal Processing 54: 2187–2197.

[20] Liu W, Pokharel PP, Principe JC (2007) Correntropy: properties and applications in non-Gaussian signal processing. IEEE Transactions on Signal Processing 55: 5286–5298.

[21] Karlen W, Brouse CJ, Cooke E, Ansermino JM, Dumont GA (2011) Respiratory rate estimation using respiratory sinus arrhythmia from photoplethysmography. In: Conference Proceedings -IEEE Engineering in Medicine and Biology Society, volume 2011: 1201–1204. 10.1109/IEMBS.2011.6090282 22254531

[22] Vapnik VN (1998) Statistical Learning Theory. New York: Wiley.

[23] Genton M (2002) Classes of kernels for machine learning: a statistics perspective. The Journal of Machine Learning Research 2: 299–312.

[24] Therrien CW (1992) Discrete Random Signals and Statistical Signal Processing. Englewood Cliffs: NJ: Prentice-Hall.

[25] Rissanen J (1978) Modeling by shortest data description. Automatica 14: 465–471.

[26] Fleming S, Thompson M, Stevens R, Heneghan C, Plüddemann A, et al (2011) Normal ranges of heart rate and respiratory rate in children from birth to 18 years of age: a systematic review of observational studies. Lancet 377: 1011–1019. 21411136 10.1016/S0140-6736(10)62226-X PMC3789232

[27] Karlen W, Turner M, Cooke E, Dumont GA, Ansermino JM (2005) CapnoBase: Signal database and tools to collect, share and annotate respiratory signals. In: Annual Meeting of the Society for Technology in Anesthesia (STA), volume 48. p. 25.

[28] Johansson A (2003) Neural network for photoplethysmographic respiratory rate monitoring. Medical & Biological Engineering & Computing 41: 242–250. 12803287 10.1007/BF02348427

[29] Patino M, Redford DT, Quigley TW, Mahmoud M, Kurth CD, et al.. (2013) Accuracy of acoustic respiration rate monitoring in pediatric patients. Paediatric Anaesthesia : In press. 10.1111/pan.12254 24033591

[30] Elgendi M, Norton I, Brearley M, Abbott D, Schuurmans D (2013) Systolic peak detection in acceleration photoplethysmograms measured from emergency responders in tropical conditions. PLoS ONE 8: e76585. 24167546 10.1371/journal.pone.0076585 PMC3805543

[31] Yang P, Dumont GA, Ansermino JM (2009) Sensor fusion using a hybrid median filter for artifact removal in intraoperative heart rate monitoring. Journal of Clinical Monitoring and Computing 23: 75–83. 19199059 10.1007/s10877-009-9163-2

